# Bactericidal Effect of Different Photochemical-Based Therapy Options on Implant Surfaces—An In Vitro Study

**DOI:** 10.3390/jcm13144212

**Published:** 2024-07-19

**Authors:** Johannes-Simon Wenzler, Svenja Caroline Wurzel, Wolfgang Falk, Sarah Böcher, Piet Palle Wurzel, Andreas Braun

**Affiliations:** 1 Department of Operative Dentistry, Periodontology and Preventive Dentistry, Rheinisch-Westfälische Technische Hochschule (RWTH) University Hospital, Pauwelsstrasse 30, 52074 Aachen, Germanyanbraun@ukaachen.de (A.B.); 2 Center for Dental Microbiology, Oro-Dental Microbiology, Hamburger Chausse 25, 24220 Flintbek, Germany

**Keywords:** dental implants, biofilm, bacterial reduction, photodynamic therapy, photothermal therapy

## Abstract

**Objectives***:* Photochemical systems are frequently recommended as an adjuvant treatment option in peri-implantitis therapy. The aim of the present study was to evaluate the efficacy of these treatment options, as well as a novel curcumin-based option, in a biofilm model on implants. **Methods***:* Eighty dental implants were inoculated with an artificial biofilm of periodontal pathogens and placed in peri-implant pocket models. The following groups were analyzed: I, photodynamic therapy (PDT); II, PDT dye; III, curcumin/DMSO + laser; IV, curcumin/DMSO only; V, dimethyl sulfoxide (DMSO) only; VI, photothermal therapy (PTT); VII, PTT dye; VIII, control. After treatment, remaining bacterial loads were assessed microbiologically using quantitative real-time polymerase chain reaction analysis. **Results***:* The PDT, PTT, and DMSO treatment methods were associated with statistically significant (*p* < 0.05) improvements in germ reduction in comparison with the other methods and the untreated control group. The mean percentage reductions were as follows: I (PDT) 93.9%, II (PDT dye) 62.9%, III (curcumin/DMSO + laser) 74.8%, IV (curcumin/DMSO only) 67.9%, V (DMSO) 89.4%, VI (PTT) 86.8%, and VII (PTT dye) 66.3%. **Conclusions***:* The commercially available PDT and PTT adjuvant treatment systems were associated with the largest statistically significant reduction in periopathogenic bacteria on implant surfaces. However, activation with laser light at a suitable wavelength is necessary to achieve the bactericidal effects. The use of curcumin as a photosensitizer for 445 nm laser irradiation did not lead to any improvement in antibacterial efficacy in comparison with rinsing with DMSO solution alone.

## 1. Introduction

Over the past 50 years, dental implants have evolved from experimental studies to become a highly predictable option for replacing missing teeth, and they now offer functional and biological advantages with good long-term success rates [1]. During this period, many changes have been introduced in relation to implant shape [2], surface [3], material, and surgery; bone augmentation, for example, has led to an expansion of the range of indications [1]. Although the achievements are impressive, intraoperative and postoperative complications may occur. Intraoperatively, the main risks are nerve injury (anesthesia, paresthesia, hypesthesia) [4] and contamination with a primarily bacterial origin, while postoperatively, inflammation around the implant after placement is the predominant complication. The most serious postoperative complication is probably loss of the implant: Quirynen et al. found that with the exception of occlusal overload, late implant loss is mostly due to bacteria as a causative factor for peri-implant diseases [5,6].

A new classification of periodontal and peri-implant diseases was introduced at the “World Workshop on the Classification of Periodontal and Peri-implant Diseases and Conditions”, a consensus conference of the American Academy of Periodontology (AAP) and the European Federation of Periodontology (EFP) that was held in Chicago in 2017 and attended by 110 experts from all over the world. Peri-implant health, as well as peri-implant diseases and conditions, were defined for the first time in the framework of this new classification [7]. Peri-implant diseases are inflammatory conditions that affect the peri-implant tissues; they are classified into peri-implant mucositis and peri-implantitis [8]. What is known as peri-implant mucositis is defined as a reversible inflammation of the soft tissue surrounding the implant; if it is allowed to persist, it can develop into peri-implantitis, as it is believed that peri-implant mucositis always precedes peri-implantitis [7,8]. Peri-implantitis, on the other hand, is characterized by inflammation of the peri-implant mucosa, accompanied by progressive loss of the supporting bone, which can ultimately result in the loss of the implant [7,8,9,10]. While peri-implant mucositis has a prevalence of approximately 80% of patients and 50% of implant sites, peri-implantitis shows a lower prevalence of 28–77% of patients and 12–43% of implant sites [9]. Peri-implant diseases, therefore, represent a growing problem for public health [8].

A new guideline on the prevention and treatment of peri-implant diseases was recently developed [8]. The overall approach to the management of peri-implant diseases is based on a combination of early detection and implementation of preventive and therapeutic interventions, which aim firstly to prevent the onset of these conditions and then treat them as early as possible to prevent progression and ultimate loss of the implant [8]. Peri-implant biofilms are considered to be the primary etiological factor for peri-implant diseases [7,8]. They form on the implant surfaces and implant-supported restorations, similar to the way in which dental plaque biofilms form on teeth [8,11,12,13]. The interventions primarily target the removal of these bacterial biofilms. Interventions for the management of peri-implant mucositis focus on supragingival biofilm control using self-performed, effective oral hygiene along with professional mechanical plaque removal, and if an implant-supported restoration does not allow for proper cleanability and modification of the restoration. As peri-implant mucositis is treatable and can lead to the reestablishment of peri-implant tissue health, the treatment of peri-implant mucositis is also the primary intervention in the prevention of peri-implantitis. Once peri-implantitis has developed, treatment will not be able to reestablish intact peri-implant tissues, even if the inflammation is successfully controlled. Disruption of the locally accumulating microbial biofilms is a key target in the treatment of peri-implantitis. As the supragingival and transmucosal restoration and abutment surfaces are specially designed to withstand biofilm formation, they are equally responsive to the same measures for infection prevention and control as natural teeth [8].

The treatment of peri-implantitis is based on the successful approaches developed for the treatment of periodontitis, aiming at the elimination of bacteria, the reduction of infection, and the creation of long-term stable conditions [14]. It includes nonsurgical therapy, followed by reevaluation and, if necessary, subsequent surgical therapy. As the main objective of peri-implantitis treatment is to control peri-implant biofilms and inflammation, the central intervention is subgingival instrumentation [8]. In view of the rough surface of the implant part that is intended for intraosseous placement, which is necessary for successful osseointegration, the removal of biofilm becomes a greater challenge if this surface is exposed and accessible for the formation of biofilm. The removal of biofilm and, above all, the complete elimination of bacteria become very difficult, if not impossible, but these are necessary for successful treatment.

Nonsurgical treatment options include mechanical cleaning of the implant with a titanium or plastic curette, sonic/ultrasonic or powder jet devices, and laser-based treatment options (e.g., Er:YAG laser). Topical antiseptic medication and laser-based treatments (e.g., disinfection using Nd:YAG lasers or diode lasers)—and in particular, diode laser-based photochemical systems such as antimicrobial photodynamic therapy (PDT) and photothermal therapy (PTT)—can be used as adjuvant treatment options [15]. Both PDT and PTT use photosensitive dyes, known as photosensitizers. After activation with laser light at a suitable wavelength, singlet oxygen is produced in the case of antimicrobial PDT [16,17], which causes oxidative damage to the cell membrane and organelles of the bacterial cells and thus has an antibacterial effect. In contrast, PTT primarily results in increased absorption of the laser light in the dye, and a local hyperthermal process then leads to the elimination of microorganisms [18].

The modern revival of interest in natural medicines has also drawn attention to curcumin, a component of the turmeric rhizome *Curcuma longa*. Various positive properties (including anti-inflammatory, antioxidant, and chemotherapeutic effects) have been attributed to it, and its use as a photosensitizer has also been evaluated [19,20]. For dental use, for example, a study on periodontitis therapy showed that curcumin gel was more effective than chlorhexidine gel as an adjuvant to conventional mechanical treatment [21]. The use of curcumin as a photosensitizer in combination with blue-wavelength light also showed promising results in some studies [22,23]. Overall, however, the research results on this are very heterogeneous. None of the studies has been able to provide clear evidence on the effectiveness of curcumin as a photosensitizer in comparison with conventional PDT and PTT methods. Another limitation is that most of the studies have not taken the influence of the organic solvent dimethyl sulfoxide (DMSO) into account, which is often used as a solvent due to the poor solubility of curcumin and which itself may contribute to bacterial reduction [19]. Although studies have previously investigated the efficacy of PDT and PTT procedures as adjuvant treatment options in the context of peri-implantitis therapy, studies describing direct comparisons are still rare. The aim of the present study was, therefore, to compare the antibacterial efficacy of the above-mentioned procedures (PDT, PTT), as well as curcumin as a photosensitizer for 445 nm laser irradiation on implant surfaces. In addition, the influence of the solvent DMSO and the necessity of activation by laser irradiation for the antibacterial effect were investigated.

## 2. Materials and Methods

### 2.1. Experimental Design

**Figure 1:**
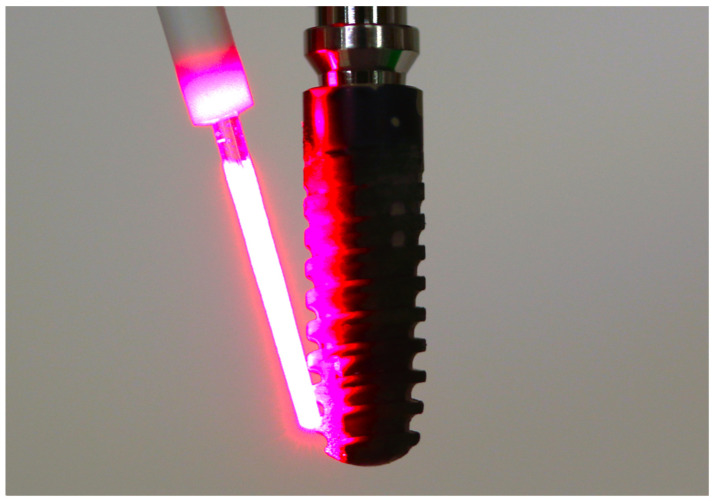
Representative image of the treatment procedure for the PDT group, with the HELBO^®^ Blue Photosensitizer and HELBO^®^ TheraLite laser with the HELBO^®^ 3D Pocket Probe (HELBO, bredent medical, Walldorf, Germany). To allow for better visualization, the image is shown without a peri-implant pocket model.

Eighty implants (Ankylos C/X Implant A 11; Dentsply Implants, Mannheim, Germany) (Figure 1) inoculated with an artificial biofilm consisting of common pathogenic periodontal bacteria such as Aggregatibacter actinomycetemcomitans (Aa), Campylobacter rectus (Cr), Eikenella corrodens (Ec), Fusobacterium nucleatum (Fn), Porphyromonas gingivalis (Pg), Prevotella intermedia (Pi), Parvimonas micra (P.m.), Treponema denticola (Td), and Tannerella forsythia (Tf) were used for this study. The preparation and inoculation of the samples were carried out by the Oro-Dental Microbiology laboratory in Kiel, Germany. For this purpose, the implants were first autoclaved with 2-propanol and then a 1.5% sodium hypochlorite solution. The process ended with draining the solution and drying the implants. Subsequently, the implants were inoculated with a bacterial suspension, as previously described by Wenzler et al. [24]. A total of 500 μL of thioglycollate suspension from a highly bacterially positive patient sample (according to qualitative PCR results with the selection criterion of as many pathogens as possible) and 500 μL of saliva from voluntary donors formed the starting medium. Saliva and bacteria were added in one step here, as the aim of this study was to “simulate” a biofilm as far as possible and not to grow it de novo. By adding saliva to the bacterial suspension, an approximately physiological matrix should be generated for the biofilm model in order to simulate a matrix similar to that of a biofilm.

For each implant, 10 µL of this bacterial suspension was pipetted onto the surface and allowed to dry at room temperature for 24 h. This procedure was repeated four times, with the implant being rotated by 90° each time so that the implants were then circularly coated with the artificial biofilm, which was subjected to visual control. The bacterial coating was limited to the part of the implant intended for intraosseous placement (screw part, apical approx. 9 mm). The artificial bacterial coating contained the common periopathogenic bacteria described above. The test specimens were then evenly distributed among all groups.

**Figure 2:**
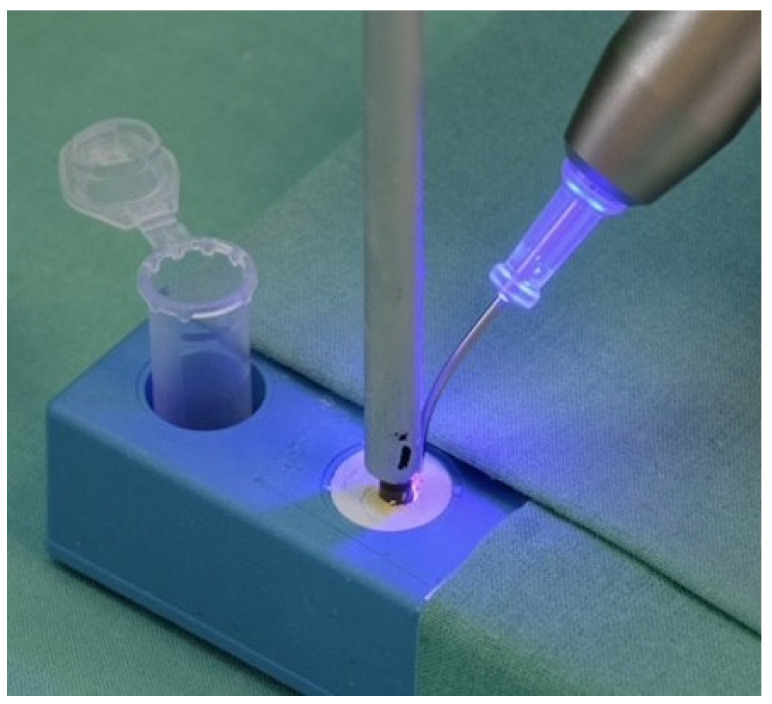
Representative image of the treatment procedure for the curcumin/DMSO + laser group (445 nm, 0.6 W, 25% duty cycle, 100 Hz, power density 186.5 mW/cm^2^; Sirona Dentsply, Bensheim, Germany).

To simulate the implant-surrounding tissue, an artificial peri-implant pocket model was created for each implant. For this purpose, a separate implant was first coated with a circular wax layer approximately 0.8 mm thick and used to fabricate negative molds or hollow bodies made of dental silicone (Flexitime easy putty, Kulzer GmbH, Hanau, Germany). The implants covered with the artificial biofilm were then each fixed in this type of peri-implant pocket model by means of an aluminum tube held by a tripod with a sleeve and clamped so that an artificial pocket/gap with a width of 0.8 mm was created around each implant (Figure 2). The test specimens placed in the simulated peri-implant pockets were then treated according to their group affiliation. The following groups were analyzed:

- PDT: HELBO^®^ Blue Photosensitizer (exposure time 180 s, followed by rinsing with 5 mL 0.9% NaCl solution; HELBO, bredent medical, Walldorf, Germany), laser treatment with the HELBO^®^ TheraLite laser (660 nm, continuous wave mode, 100 mW, power density 70.74 W/cm^2^), and the HELBO^®^ 3D Pocket Probe—with application at six points per specimen for 10 s each;
- PDT dye: HELBO^®^ Blue photosensitizer without laser application (exposure time 180 s);
- Curcumin/DMSO + laser: staining of microorganisms with a mixed curcumin solution (100 mg/L (Merck KGaA, Darmstadt, Germany); organic solvent: 0.5 mL DMSO (Sigma Aldrich, St. Louis, MO, USA) as an emulsifier in the form of a 0.5% DMSO solution). After an exposure time of 180 s, the samples were rinsed with 5 mL of 0.9% NaCl solution. Laser treatment with the SIROLaserBlue (445 nm, 0.6 W, 25% duty cycle, 100 Hz, power density 373.02 W/cm^2^; Sirona Dentsply, Bensheim, Germany) with a 320-µm EasyTip fiber—with application at six points per specimen for 10 s each;
- Curcumin/DMSO only: curcumin/DMSO solution only (see III) without laser application (exposure time 180 s);
- DMSO: DMSO solution (0.5%) only, without laser application (exposure time 180 s);
- PTT: EmunDo^®^ dye was applied (exposure time 180 s, followed by rinsing with 5 mL 0.9% NaCl solution) and laser treatment with a FOX Q810plus laser (810 nm, continuous wave, effective power 200 mW, power density 565.90 W/cm^2^; EmunDo^®^, A.R.C. Laser, Nuremberg, Germany) was carried out—with application at six points per specimen for 10 s each;
- PTT dye: EmunDo^®^ dye without laser application (exposure time 180 s);
- Control: untreated control group.

The application of the different therapy options was carried out using a standardized protocol by a single practitioner. The processed specimens were then removed from the peri-implant pocket models, and all rinsed with a 5 mL sterile NaCl solution. They were then placed in Eppendorf tubes and sent for microbiological analysis.

### 2.2. Microbiological Analysis

The samples were analyzed in a microbiology laboratory (Oro-Dentale Mikrobiologie ODM, Kiel, Germany) using quantitative real-time polymerase chain reaction (qPCR) after DNA extraction and quantification of the microbiological samples. The main parameter for analysis was the total bacterial load (TBL), expressed in genome-equivalent colony-forming units (CFUs) per milliliter in accordance with internal laboratory standards.

### 2.3. Statistical Analysis

A power analysis was performed prior to this study. For this purpose, the effect size was set to 0.5 in accordance with Cohen [25]. For an alpha error of 0.05 and a power of 0.8, a sample size of 10 in each group was calculated. The normal distribution of the values was tested using the Shapiro–Wilk test. The Kruskal–Wallis and Mann–Whitney tests were used to compare the bacterial counts as a function of the experimental groups. These are tests for unconnected samples in which a *p* value of *p* > 0.05 indicates that there is no statistically significant difference.

## 3. Results

It was found that the quantity of remaining bacteria on the implant surfaces was dependent on the treatment method used. A statistically significant reduction in the total bacterial load (TBL) was observed in three experimental groups (I, V, VI; *p* < 0.05), with the lowest bacterial counts observed in group I (PDT), with a median value of 2.81 × 10^7^ colony-forming units (CFUs) (min. 8.19 × 10^6^, max. 5.31 × 10^7^, interquartile range [IQR] 2.52 × 10^7^), followed by group V (DMSO), with a median value of 2.91 × 10^7^ CFU (min. 1.51 × 10^7^, max. 7.31 × 10^7^, IQR 3.67 × 10^7^), and group VI (PTT), with a median value of 3.56 × 10^7^ CFU (min. 1.06 × 10^7^, max. 1.19 × 10^8^, IQR 4.19 × 10^7^). All three groups showed statistically significant differences from the other study groups (*p* < 0.05) but not among each other (*p* > 0.05).

With the exception of group II (PDT dye), with a median value of 1.52 × 10^8^ CFU (min. 1.46 × 10^7^, max. 3.21 × 10^8^, IQR 1.82 × 10^8^), and group VII (PTT dye), with a median value of 1.26 × 10^8^ CFU (min. 1.48 × 10^7^, max. 4.43 × 10^8^, IQR 2.34 × 10^8^; *p* > 0.05), all the other groups also showed a statistically significant difference in terms of TBL reduction in comparison with group VIII (control), with a median value of 4.21 × 10^8^ CFU (min. 1.16 × 10^8^, max. 8.38 × 10^8^, IQR 6.00 × 10^8^; *p* < 0.05).

**Figure 3:**
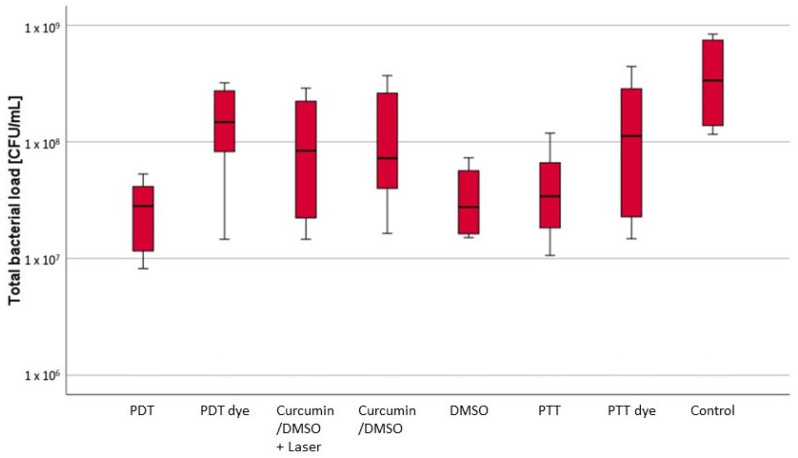
Box plot diagram for the total bacterial load (TBL) in the different groups. The results of the Mann-Whitney test can be found in Table 2.

The groups based on the curcumin/DMSO solution with (III) and without laser irradiation (IV) did not show any statistically significant differences between each other (*p* > 0.05), with a median value of 8.78 × 10^7^ CFU (min. 1.46 × 10^7^, max. 2.89 × 10^8^, IQR 1.81 × 10^8^) in group III (curcumin/DMSO + laser) and a median value of 7.37 × 10^7^ CFU (min. 1.64 × 10^7^, max. 3.70 × 10^8^, IQR 1.78 × 10^8^) in group IV (curcumin/DMSO only) (Figure 3, Table 1 and Table 2).

The percentage reductions of the total bacterial load—i.e., the bacterial reduction (in %) in the individual test groups in comparison with the untreated control group—were as follows: I (PDT) 93.9%, II (PDT dye) 62.9%, III (curcumin/DMSO + laser) 74.8%, IV (curcumin/DMSO only) 67.9%, V (DMSO) 89.4%, VI (PTT) 86.8%, and VII (PTT dye) 66.3%.

## 4. Discussion

In principle, the effectiveness of mechanical debridement, which is regarded as the gold standard in periodontitis and peri-implantitis therapy, can be impaired by morphological conditions on the subgingival tooth surface and, in particular, by the complex macrostructure and microstructure of implant surfaces [26,27]. Due to these limitations, the desired endpoint of therapeutic interventions—complete bacterial elimination (or at least extensive reduction)—is very difficult, if not impossible. Several adjuvant therapies have, therefore, been proposed to achieve further bacterial reduction—for example, the PDT and PTT approaches [28]. Mechanical treatment procedures also harbor a risk of unintentional release of titanium particles, especially if the implants are incorrectly cleaned during subgingival debridement using manual or sonic/ultrasonic procedures. Here too, photochemical therapy procedures could be advantageous [29,30]. In the present study, the commercially available adjuvant therapy systems PDT (I) and PTT (VI), along with the DMSO group (V), were associated with statistically significant differences (*p* < 0.05) from the control group and the greatest bacterial reduction in comparison with the other study groups. Generally, the nomenclature and differentiation of photodynamic and photothermal reactions and systems are often difficult. In the literature, the term “photochemical reactions” is often used as a generic term for both photodynamic and photothermal reactions. Other authors use the generic term “photodynamic reactions” for photochemical and photothermal reactions. In principle, these terms refer to the combination of a photosensitive dye and a light source at a suitable wavelength to activate it; the reaction is then mediated via different mechanisms by releasing reactive oxygen species (as in the PDT system used here, for example) or via a local hyperthermal reaction (as in the PTT system used in the present study, for example). The term “photochemical reactions” is used as the generic term in the present study.

The results of this study showed a statistically significant reduction (*p* < 0.05) in the TBL in groups I (PDT) and VI (PTT). The results are, therefore, consistent with previous findings on the antibacterial efficacy of PDT and PTT [31,32]. To ensure greater accuracy and to investigate the need for activation by means of laser light, each component (dye, solvent, and combination) was also examined separately to rule out the possibility that the individual components might have antimicrobial effects on their own. In this respect, the PDT and PTT groups showed a statistically significantly (*p* < 0.05) greater reduction in the TBL than the groups without laser activation of the dyes, which showed only a small but statistically nonsignificant difference (*p* > 0.05) from the control group. This is an important point regarding which these systems are often criticized. Some have postulated that the photosensitizer alone mediates the antibacterial effect and that irradiation with laser light is not actually necessary. However, the results of the present study underline the necessity for the dyes to be activated with laser light in order to achieve an antibacterial effect. This result is also consistent with the results of a previous study by Schneider et al., who found that laser irradiation is an essential part of antimicrobial photodynamic therapy to reduce bacteria [33]. Thus, for the groups without laser activation of the photosensitizer (II and VII), it can be assumed that no effect in terms of TBL reduction was achieved due to the lack of activation by the corresponding laser system [19,20]. The slight but not statistically significant differences (*p* > 0.05) from the control group can probably be attributed to the removal/rinsing of the photosensitizer solution by rinsing it with a saline solution (NaCl), which appears to cause a small change in TBL by simply rinsing away some bacteria. This effect has already been discussed in other studies [19,24]. One point of criticism here is that the control group (VIII) was not subjected to analogous rinsing with a saline solution, which might have ensured better comparability. However, in view of the only marginal influence of this additional rinsing, a significant influence on the results is not to be expected here.

On the other hand, curcumin/DMSO activated by laser light (III) did not show a greater reduction in TBL than curcumin/DMSO without laser activation (IV), so no improvement in antibacterial efficacy was observed in the present study when curcumin was used as a photosensitizing agent for the 445 nm laser. This finding is consistent with the results of a previous study by Böcher et al., who also did not observe any improved antibacterial efficacy with curcumin activated by laser light [19]. Other studies, such as Quishida et al., reported a substantial reduction in the total biomass of a multispecies biofilm for a curcumin solution activated by LED light [19,34]. As previous studies had already reported increased antibacterial efficacy with curcumin activated with laser light [34], the study by Böcher et al. concluded that the wavelength of 445 nm used may not have been suitable and, therefore, did not result in any improvement. They, therefore, hypothesized that curcumin may have a narrow absorption range in the blue spectrum [19]. A recent systematic review by Etemadi et al., which included both in vitro studies and clinical trials, concluded that curcumin as a photosensitizer activated by a blue wavelength is effective in eliminating the various bacterial species involved in periodontal disease but that more clinical studies are needed to identify the best protocol (including the suitable concentration of curcumin, a suitable solvent, and appropriate laser parameters) [35].

Groups III and IV, with the combined curcumin and DMSO solution—with (III) or without (IV) laser irradiation—as well as group V (DMSO alone), also showed statistically significant differences (*p* < 0.05) in TBL reduction in comparison with the control group. On the other hand, curcumin/DMSO activated by laser light (III) did not show a greater reduction in TBL than curcumin/DMSO (IV) or DMSO alone (V), suggesting that the bacterial reduction may be due to the emulsifier DMSO alone. In addition, the groups with the combined curcumin and DMSO solution—with (III) or without (IV) laser irradiation—showed a slight but statistically nonsignificantly (*p* > 0.05) lower bacterial reduction in comparison with the group with the DMSO solution alone (V), which may indicate that the antibacterial efficacy of DMSO is somewhat attenuated when it is combined with curcumin, with the associated reduction in its concentration. DMSO is an organic solvent that promotes the penetration of dissolved substances and is described as a pharmacological agent and a free radical scavenger. It is also reported to have antimicrobial activity and anti-inflammatory effects. In vitro, DMSO showed bacteriostatic and bactericidal activity against staphylococci, streptococci, and other bacteria at concentrations of 5% and more [36]. In vivo, DMSO has been shown to reduce the population of oral flora [37] and is considered to have anti-inflammatory action through effects on the immune response and as a free radical scavenger [36]. However, the effects of DMSO described appear to be somewhat weakened in vivo, which is particularly important for future application in patients [36]. Previous studies have reported that curcumin-based rinsing solutions show promising results in relation to the reduction in periodontal and peri-implant pathogenic bacteria [38,39]—a finding that is consistent with the results of the curcumin/DMSO-based groups III and IV in the present study—although the influence of the solvent DMSO was often not taken into account. Only one other study by our own research group, by Böcher et al., has investigated the influence of the emulsifier DMSO itself and also found that a curcumin/DMSO solution with or without additional laser application did not have a greater antibacterial potential than DMSO alone [19], a finding that is also consistent with the present study.

However, several factors in the experimental setup can significantly influence the results when in vitro studies are compared. These include the effect of the solvent used, as well as the parameters of the application or laser activation, the experimental setup, the artificial biofilm, etc.—making comparison very difficult, if not impossible. For example, some studies do not report at what concentration the curcumin solution was applied or which solvent was used. In some studies in which details are reported, ethanol, sterile water, or saline were used instead of DMSO [19,40,41,42], and also the curcumin concentration used differed widely [41,43]. This is particularly important, as curcumin may have a specific absorption spectrum depending on the concentration used [44]. The results of the present study also indicate that the absorption spectrum of the concentration of curcumin solution used has not been appropriate and that there is a need for optimization on these points.

In addition to the previously described antibacterial effects that DMSO alone can have, blue light alone is also reported to have a decisive effect. Studies have reported that blue light sources have phototoxic effects on periopathogenic bacteria and that phototherapy, even when used alone, can lead to a significant reduction in biofilm growth [19,35,45,46,47]. In endodontics, LED-based light sources or lasers, such as the 445 nm laser, have also been shown to have bactericidal effects on endodontic bacteria [48,49,50]. As the bacterial complexes in the two fields of periodontology and endodontology overlap, similar effects on the bacteria can also be assumed here [51]. This again underlines the importance of investigating the individual components separately in order to ensure maximum accuracy, and it may be a limitation of the present study, as a group with laser application alone was not included here. Consequently, any reduction in bacterial counts achieved may be due to a combination of different effects, and further systematic investigations are required.

Antimicrobial photochemical therapy systems using diode lasers have been the subject of numerous studies investigating the effect of different photosensitization systems on different periopathogenic microorganisms or as a therapeutic option in vivo. Antimicrobial photodynamic therapy (PDT) using phenothiazine chloride (as in the PDT system used in the present study) is now a widely used and well-studied therapeutic method. In dentistry, it is mainly used in periodontitis and peri-implantitis therapy. Studies have shown promising results, particularly in relation to the bactericidal effects of PDT procedures [52,53,54]. On implant surfaces, studies have also reported effective adjuvant bacterial reduction [19,55,56,57] and promising results with regard to clinical parameters, although only with short-term follow-up periods of up to 12 months [58,59,60]. The present results further support these findings, as the PDT group (I) showed the best results in terms of TBL reduction, along with the PTT (VI) and DMSO (V) groups. It can, therefore, be assumed that PDT (also as an adjuvant therapy option) can achieve additional bacterial reduction. However, further well-controlled clinical trials (with longer follow-up periods) are still necessary in order to further validate these good results. These studies will need to ensure that the PDT is performed correctly (with the corresponding dye and wavelength) and is repeated as an adjuvant measure at the recommended regular treatment intervals. Unfortunately, many studies on photochemical therapy options are poorly conducted (in relation to the choice of an unsuitable dye or wavelength, comparison of different wavelength ranges, incorrect application modalities, etc.), and their conclusions are, therefore, not scientifically valid. However, the limitations of photochemical systems should also be taken into account. As PDT is an oxygen-dependent procedure, its efficacy may be compromised by decreasing oxygen availability, such as in deep periodontal pockets or deep peri-implant defects, as well as in deeper biofilm layers [45,61]. The PTT system used here as a representative of photothermal therapy is based on indocyanine green as a photosensitive dye (EmunDo^®^). Its mechanism of action is primarily based on photothermal effects that result in a local hyperthermal reaction, which is then responsible for eliminating the bacteria [62]. The occurrence of photodynamic side effects due to the appearance of singlet oxygen has also been reported in this context. In contrast to photodynamic reactions, however, this mainly oxidizes the molecule itself and leads to further degradation [61]. As an advantage of this procedure, the substantial independence of oxygen (in contrast to PDT) can be mentioned; this could be particularly beneficial in deep periodontal or peri-implant defects. However, potential thermal damage to the surrounding tissue should be taken into account here [61,63,64]. The antibacterial efficacy of indocyanine green activated with laser light at a wavelength of 800–810 nm has already been demonstrated in vitro. Clinical studies in the field of periodontology have also shown improvements in some clinical parameters involving the reduction of periodontal pathogens [65,66]. However, only a limited number of studies on PTT are available to date. According to the initial results, it appears to be a promising approach for the treatment of bacteria-related peri-implant diseases, but further research is required here [66].

Further limitations of photochemical systems should be mentioned here, as some studies have also reported ambiguous results [67]. These may be related to the greater complexity of the biofilm structure in vivo. Since bacteria that are organized in biofilms have been shown to be less accessible to antibiotic substances due to protection within the extracellular polymer matrix, it should be noted that the uptake of photosensitizers is similarly restricted [15,68,69]—so that effective penetration and, above all, antibacterial efficacy may be impaired. The composition and pH of the surrounding fluid also influence the effectiveness of PDT procedures [70]. However, due to widely varying study designs, the results of in vitro studies, in particular, are limited and should be treated with caution; direct extrapolation to actual in vivo conditions is hardly possible. Both PDT and PTT are described as adjuvant therapy options, as the uptake of the photosensitive dyes may be impeded by an intact biofilm in the same way as has been described for the uptake of antibiotic substances. Disruption of the biofilm is, therefore, regarded as a prerequisite. This can be considered a limitation of the present study, as the two approaches were not used for the purpose of adjuvant therapy here since no mechanical debridement was performed initially. However, as PDT has also been reported to cause the breakdown of extracellular polymer substances and the accessibility of and, therefore, the ability to disrupt the biofilm is limited, particularly in the area between the screw threads of implants, the aim of the present study was to investigate the influence of these systems in a “worst-case scenario”—i.e., when the biofilm is not disturbed but completely intact. As a reduction in bacteria was also demonstrated on this “intact” and “undisturbed” biofilm, it can be assumed that an even greater antibacterial reduction can be achieved on previously disturbed biofilm. The recommendation that biofilms should be mechanically disrupted prior to the adjuvant application of photochemical systems, of course, remains unaffected.

In general, it must be taken into account that a certain degree of inaccuracy can always occur during clinical application by the practitioner as a human component, resulting in not all areas of the implant surface being reached evenly. Such differences can be addressed in a study by having a single user carry out the entire practical part. This makes it possible to assume the same conditions in this study, at least intra-experimentally.

## 5. Conclusions

The commercially available PDT and PTT adjuvant treatment systems were associated with the statistically significantly greatest reduction in periopathogenic bacteria on implant surfaces. Activation with laser light at a suitable wavelength is, however, necessary to achieve the bactericidal effects. The use of curcumin as a photosensitizer for 445 nm laser irradiation did not lead to any improvement in antibacterial efficacy in comparison with DMSO solution alone.

## Figures and Tables

**Table 1:** Total bacterial load in CFU/mL in the different study groups.

	I (PDT)	II (PDT Dye)	III (Curcumin/ DMSO + Laser)	IV (Curcumin/ DMSO Only)	V (DMSO)	VI (PTT)	VII (PTT Dye)	VIII (Control)
Mean	2.92 × 10^7^	1.64 × 10^8^	1.20 × 10^8^	1.33 × 10^8^	3.60 × 10^7^	4.76 × 10^7^	1.69 × 10^8^	4.42 × 10^8^
Standard deviation	1.65 × 10^7^	1.14 × 10^8^	1.06 × 10^8^	1.34 × 10^8^	2.23 × 10^7^	3.71 × 10^7^	1.57 × 10^8^	3.24 × 10^8^
Median	2.81 × 10^7^	1.52 × 10^8^	8.78 × 10^7^	7.37 × 10^7^	2.91 × 10^7^	3.56 × 10^7^	1.26 × 10^8^	4.21 × 10^8^
Minimum	8.19 × 10^6^	1.46 × 10^7^	1.46 × 10^7^	1.64 × 10^7^	1.51 × 10^7^	1.06 × 10^7^	1.48 × 10^7^	1.16 × 10^8^
Maximum	5.31 × 10^7^	3.21 × 10^8^	2.89 × 10^8^	3.70 × 10^8^	7.31 × 10^7^	1.19 × 10^8^	4.43 × 10^8^	8.38 × 10^8^
Interquartile range	2.52 × 10^7^	1.82 × 10^8^	1.81 × 10^8^	1.78 × 10^8^	3.67 × 10^7^	4.19 × 10^7^	2.34 × 10^8^	6.00 × 10^8^
*n*	10	10	10	10	10	10	10	10

**Table 2:** Mann–Whitney test results. Statistically significant differences were considered for *p* < 0.05 (grey fields).

	I (PDT)	II (PDT Dye)	III (Curcumin/ DMSO + Laser)	IV (Curcumin/ DMSO Only)	V (DMSO)	VI (PTT)	VII (PTT Dye)	VIII (Control)
I (PDT)		0.007285	0.0539	0.02113	0.4727	0.4727	0.03121	0.0001827
II (PDT dye)	0.007285		0.4495	0.6232	0.02113	0.04117	0.9698	0.07566
III (curcumin/DMSO + laser)	0.0539	0.4495		0.7913	0.08897	0.1859	0.5708	0.02113
IV (curcumin/DMSO only)	0.02113	0.6232	0.7913		0.03121	0.1859	0.7337	0.009108
V (DMSO)	0.4727	0.02113	0.08897	0.03121		0.4727	0.0539	0.0001827
VI (PTT)	0.4727	0.04117	0.1859	0.1859	0.4727		0.1304	0.0002461
VII (PTT dye)	0.03121	0.9698	0.5708	0.7337	0.0539	0.1304		0.06402
VIII (control)	0.0001827	0.07566	0.02113	0.009108	0.0001827	0.0002461	0.06402	

## Data Availability

The data presented in this study are available on request from the senior author (A.B.).
